# Automatic Spine Tissue Segmentation from MRI Data Based on Cascade of Boosted Classifiers and Active Appearance Model

**DOI:** 10.1155/2018/7952946

**Published:** 2018-04-29

**Authors:** Dominik Gaweł, Paweł Główka, Tomasz Kotwicki, Michał Nowak

**Affiliations:** ^1^Chair of Virtual Engineering, Poznań University of Technology, 60-965 Poznań, Poland; ^2^Department of Spine Disorders and Pediatric Orthopedics, University of Medical Sciences, 61-545 Poznań, Poland

## Abstract

The study introduces a novel method for automatic segmentation of vertebral column tissue from MRI images. The paper describes a method that combines multiple stages of Machine Learning techniques to recognize and separate different tissues of the human spine. For the needs of this paper, 50 MRI examinations presenting lumbosacral spine of patients with low back pain were selected. After the initial filtration, automatic vertebrae recognition using Cascade Classifier takes place. Afterwards the main segmentation process using the patch based Active Appearance Model is performed. Obtained results are interpolated using centripetal Catmull–Rom splines. The method was tested on previously unseen vertebrae images segmented manually by 5 physicians. A test validating algorithm convergence per iteration was performed and the Intraclass Correlation Coefficient was calculated. Additionally, the 10-fold cross-validation analysis has been done. Presented method proved to be comparable to the physicians (FF = 90.19 ± 1.01%). Moreover results confirmed a proper algorithm convergence. Automatically segmented area correlated well with manual segmentation for single measurements ($\bar{r}=0.8336$) and for average measurements ($\bar{r}=0.9068$) with *p* = 0.05. The 10-fold cross-validation analysis (FF = 91.37 ± 1.13%) confirmed a good model generalization resulting in practical performance.

## 1. Introduction

Pathology of the intervertebral disk is one of the common causes of pain in the lumbar spine. In 40% of cases, pain of the lumbosacral spine is diagnosed as a discogenic [1]. What is more, 80% of the general population will have or already have had pain of the lumbosacral spine [2–4], in 5–10% of them a chronic pain develops [1, 5].

In contemporary diagnostics Magnetic Resonance Imaging (MRI) is the modality of choice for intervertebral disc visualization. Magnetic Resonance Imaging, for almost all spinal disorders, provides robust images of the spine [6] with high quality soft-tissue visualization, much more detailed than results obtained with other modalities [7]. The additional advantage of the MRI is the lack of radiation.

Automatic tissue segmentation from Magnetic Resonance Imaging data is a challenging task, because the quality of the data affects the process; what is more, the differences between medical facilities, used protocols, and imaging machines force the necessity of universality.

Till now multiple approaches have been presented. Dong and Zheng in [8] divided the common solutions into methods that rely on graphical model [9], probabilistic model [10], watershed algorithm [11], atlas registration [12], graph cuts [13], Statistical Shape Model [14], anisotropic oriented flux [15], and random forest regression and classification [16].

The methods mentioned above are based on discrete classification returning a limited and inaccurate information about the tissue. The paper describes a method that combines multiple stages of Machine Learning (ML) [17] techniques to recognize and separate different tissues of the spine.

The objective of this study is to introduce a novel method for automatic segmentation of vertebral column tissue from MRI images.

## 2. Materials and Methods

### 2.1. The Data

For the needs of this paper 50 MRI examinations presenting lumbosacral (LS) spine of patients with low back pain were selected. The examinations were made with Siemens MAGNETOM Spectra 3T MR device. For vertebral body recognition T1 TSE (Turbo Spin Echo) Sagittal sequences, with Echo Time 9.3 ms and Repetition Time ranging from 550 ms to 700 ms, were chosen. The image sets consisted of 17 to 31 images with 4 mm slice thickness, 4.8 mm slice distance, and 384 × 384 px resolution.

### 2.2. General Procedure for Segmentation

**Figure 1:**
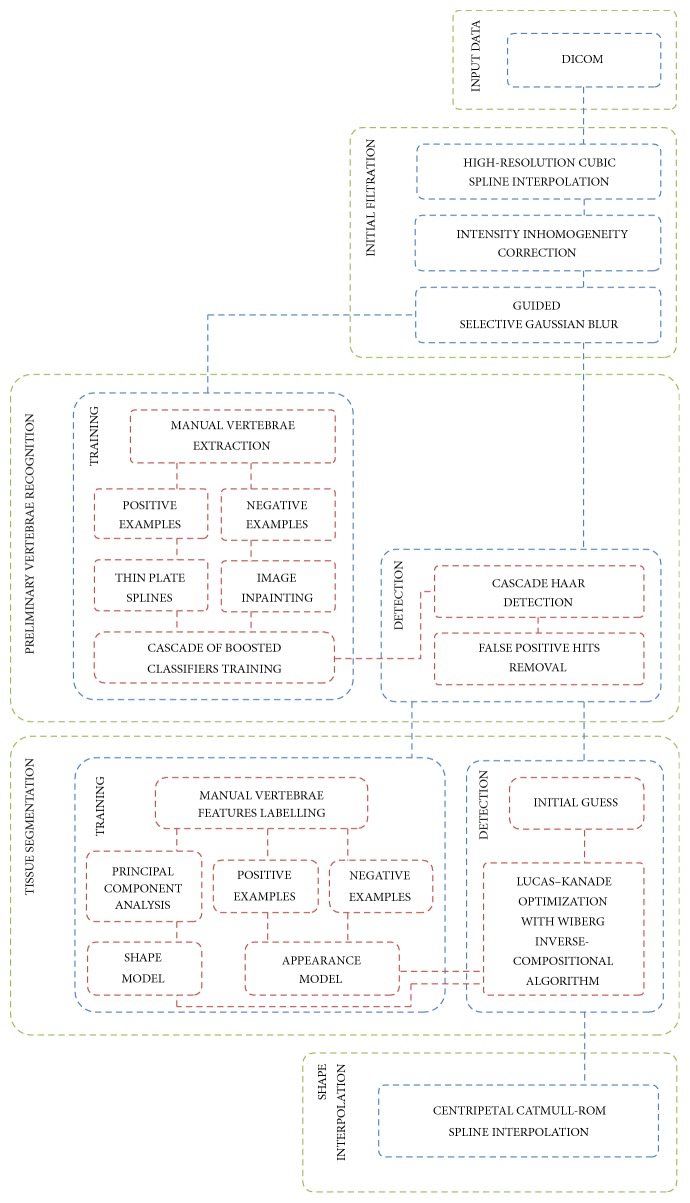
Flow chart of presented method. The solution is based on Machine Learning techniques combining Cascade of Boosted Classifiers with patch based Active Appearance Model and Principal Component Analysis.

Presented solution is based on well-known Machine Learning (ML) [18] techniques combining Cascade of Boosted Classifiers [19–21] with patch based Active Appearance Model (AAM) [22, 23] algorithm and Principal Component Analysis (PCA) [24] (Figure 1).

At the beginning DICOM images are read. After that initial filtration is made to increase the quality of the data. Afterwards automatic vertebrae recognition using Cascade Classifier [21, 25] takes place. After the initial recognition the main tissue segmentation process is made using the patch based Active Appearance Model [22, 23]. Combined information about location, shape, and appearance provides a high quality model used for search and extraction of desired tissue. The results are afterwards interpolated using centripetal Catmull–Rom splines [26–28].

### 2.3. Initial Filtration

**Figure 2:**
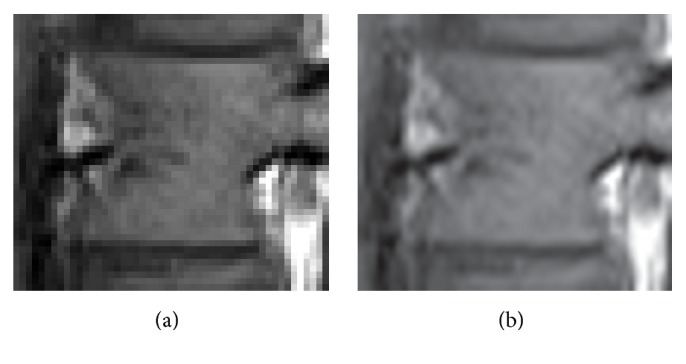
(a) Magnified image presenting sagittal slice of a single vertebra extracted from the input data. (b) The same image after initial resizing using a high-resolution cubic spline.

Due to low quality of the data (low resolution, intensity inhomogeneity, and high noise), initial filtration is needed. At the beginning the images are being resized to increase resolution. For the needs of presented method a high-resolution cubic spline has been chosen [29] (Figure 2).

**Figure 3:**
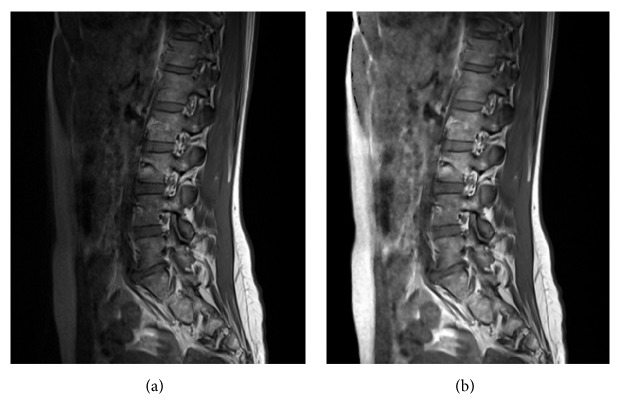
Initial filtration by recalculating local intensities to fit the global exponential function defined from boundary fat-skin tissue intensity contrast. (a) Image before IIH compensation. (b) Image after IIH compensation.

After that the developed intensity inhomogeneity (IIH) correction method is performed. The method is based on recalculating local intensities in such a way to fit the global exponential function defined from the boundary fat-skin tissue intensity contrast. After calculations a nonlinear selective Gaussian Blur [30] using the same global exponential function for parameterization is performed to remove the noise amplified through the correction process. As a result of this method, an intensity inhomogeneity correction is achieved (Figure 3).

### 2.4. Preliminary Vertebrae Recognition

At the beginning, to achieve accurate segmentation results and reduce number of MRI examinations needed for training, vertebrae recognition is made. The goal of this action is to extract each vertebra from the whole image containing spine MRI examination. To achieve this the Machine Learning [18] training of Cascade of Boosted Classifiers [19–21] based on extended set Haar-like features [31] was made.

The vertebrae recognition consists of two major stages: training the classifier and vertebrae detection. Both were done using OpenCV library [25]. For the training two types of information are needed: positive examples presenting desired object that one is looking for and negative examples presenting background. To prepare the data, special software allowing fast cutting, artificial data generation, and automatic background reconstruction was developed. For the training process 50 MRI examinations were used. From those examinations over 1000 vertebrae images were extracted manually and used for automatic creation of 10,000 artificial positive examples with Thin Plate Splines (TPS) transformations [32, 33]. Afterwards negative examples were reconstructed from the same examinations by covering previously cut out vertebrae using Image Inpainting method [34].

**Figure 4:**
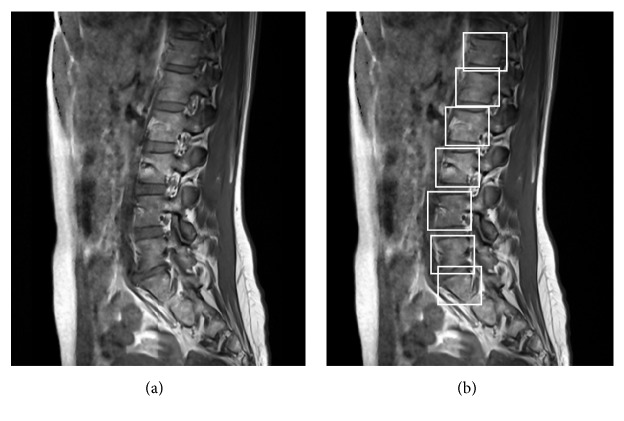
Vertebrae recognition using Cascade of Boosted Classifiers based on extended set of Haar-like features. Classifiers training based on the AdaBoost algorithm. (a) Initial image. (b) Positively detected vertebrae marked with bounding boxes for visualization.

Both positive and negative examples are afterwards used for classifiers training based on the AdaBoost algorithm [35]. Multiple weak classifiers are then combined in a cascade resembling Decision Tree [36] creating a strong classifier [37]. To achieve best performance, after the recognition, additional size constraints were introduced, removing the false positive hits. Obtained model allows proper vertebrae recognition (Figure 4).

### 2.5. Tissue Segmentation

**Figure 5:**
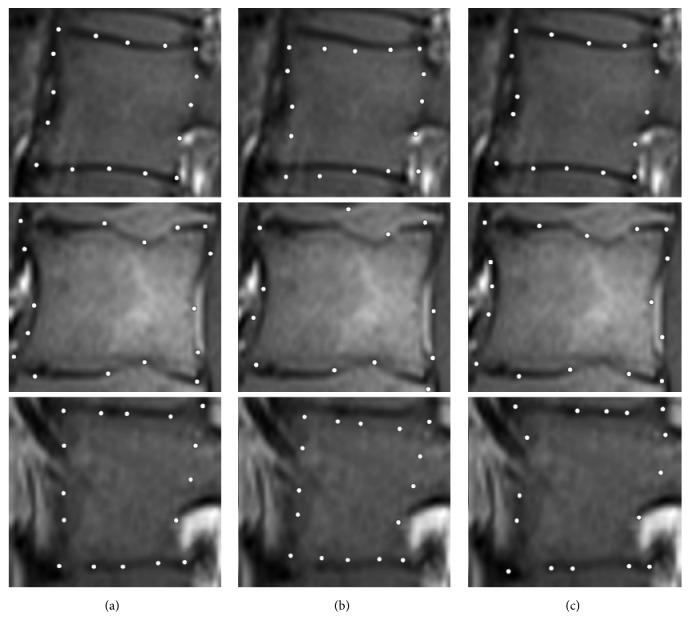
Example of landmark localization results for three different slices. (a–c) Ground truth shape, initial guess, and final result.

After the vertebrae recognition the main tissue segmentation is made. The solution is based on Active Appearance Model (AAM) [22, 23, 38] algorithm and combines a Statistical Shape Model based on Principal Component Analysis [39], with a gray-level Appearance Model. The method focuses on recognizing the predefined characteristic features from previously extracted vertebrae images by combining the information about each pretrained characteristic feature appearance with the information about features' mean position, their arrangement, and possible deviation. Similarly to preliminary vertebrae recognition, the tissue segmentation procedure consists of two stages: training and detection; however, contrary to previously trained classifiers, the built model is used for recognition of small patches instead of a whole vertebra. Each image used for the training originates from the prepared vertebrae database and was previously manually labeled by the group of five experts (physicians trained in MRI images assessment) with 16 characteristic points corresponding to vertebra features. Introduced information is used for building Point Distribution Model and creating training examples for the Appearance Model. The Point Distribution Model is used in a PCA [39] analysis to obtain the Shape Model containing information about the mean shape, eigenvectors, and eigenvalues. The positive and negative training examples are used for training to obtain the Appearance Model. Trained AAM model is afterwards used for spine tissue detection and classification. The detection procedure starts with an initial guess based on a perturbed ground truth shape. For this study a patch based AAM approach [22, 23, 38] has been chosen, representing the appearance of features as a rectangular patches distincted around each landmark. Finally optimization of the cost function is solved by Lucas–Kanade Optimization [40, 41] method with Wiberg Inverse Compositional algorithm [42–44] (Figure 5).

### 2.6. Shape Interpolation

**Figure 6:**
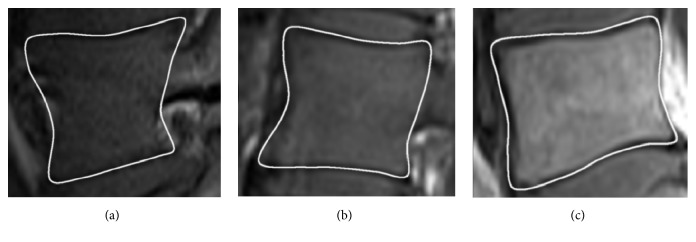
Information between the points interpolated with centripetal Catmull–Rom splines, ensuring C1 continuity, proper tightness with no self-intersubsubsections, and knot parameterization, leaving an area for further curve optimization. (a–c) Examples of a lateral, intermediate, and central part of the vertebral body.

Automatically extracted 16 feature points for each vertebra image visible in the MRI examination are afterwards used for spine tissue segmentation. The information between the points is interpolated with centripetal Catmull–Rom Splines [26–28] (Figure 6), ensuring C1 continuity, proper tightness with no self-intersubsubsections, and knot parameterization, leaving an area for further curve optimization.

## 3. Results

The method was tested on a set of 50 previously unseen vertebrae images. The spine tissue was manually segmented by 5 physicians and compared with Machine Learning results. For the numerical evaluation three measures were used [45–47]: True Positive Fraction (TPF) (1), False Negative Fraction (FNF) (2), and False Fraction (FF) (3):

(1) $$\begin{array}{c} \text{TPF (True Positive Fraction)}=\frac{A_{TP}}{A_{T}}⟶Sensitivity, \end{array}$$

where true positive area *A*_TP_ = *A*_*S*_∩*A*_*T*_, *A*_*T*_ is a manually segmented (by an expert) tissue area and *A*_*S*_ is an automatically segmented (by a computer) area.

(2) $$\begin{array}{c} \text{FNF (False Negative Fraction)}=\frac{A_{FN}}{A_{T}}⟶\\ Specificity, \end{array}$$

where false negative area *A*_FN_ = *A*_*T*_ − *A*_*S*_.

(3) $$\begin{array}{c} \text{FF (False Fraction)}=1-\frac{A_{FP}+A_{FN}}{A_{T}}⟶Accuracy, \end{array}$$

where false positive area *A*_FP_ = *A*_*S*_ − *A*_*T*_.

To achieve better performance five different optimization algorithms, available in Menpo Framework [22, 38], were tested.

**Figure 7:**
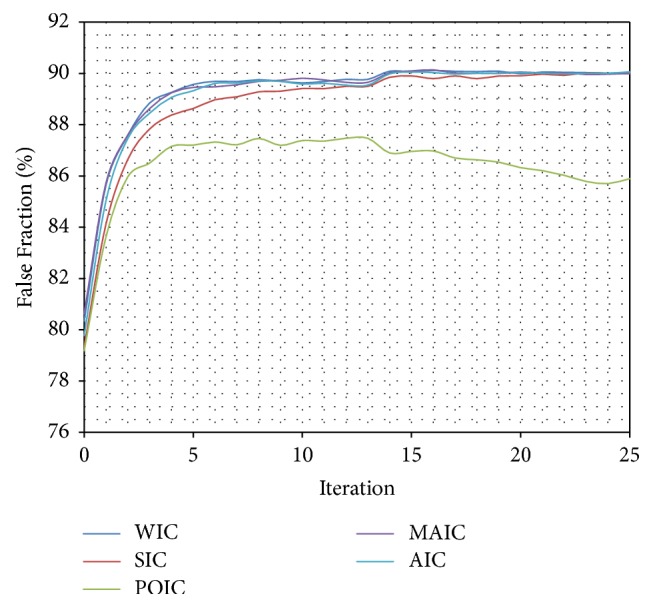
Comparison (percentage) of False Fraction mean values for subsequent interactions of automatic segmentation for different optimization algorithms.

**Figure 8:**
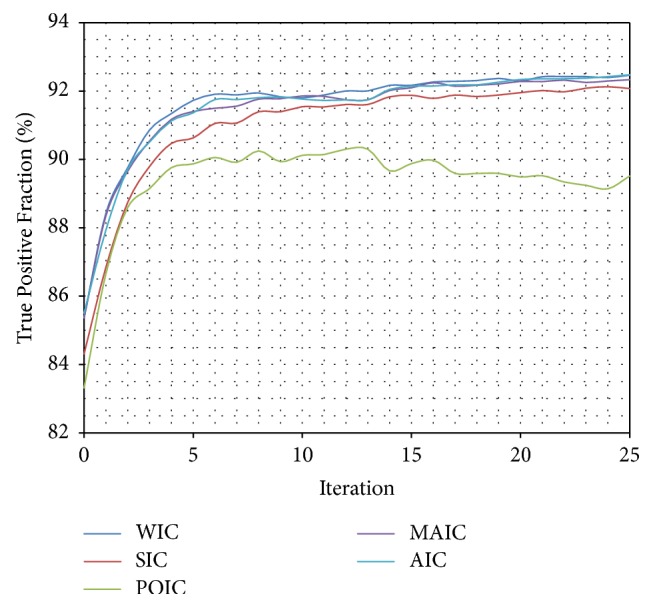
Comparison (percentage) of True Positive Fraction mean values for subsequent interactions of automatic segmentation for different optimization algorithms.

**Figure 9:**
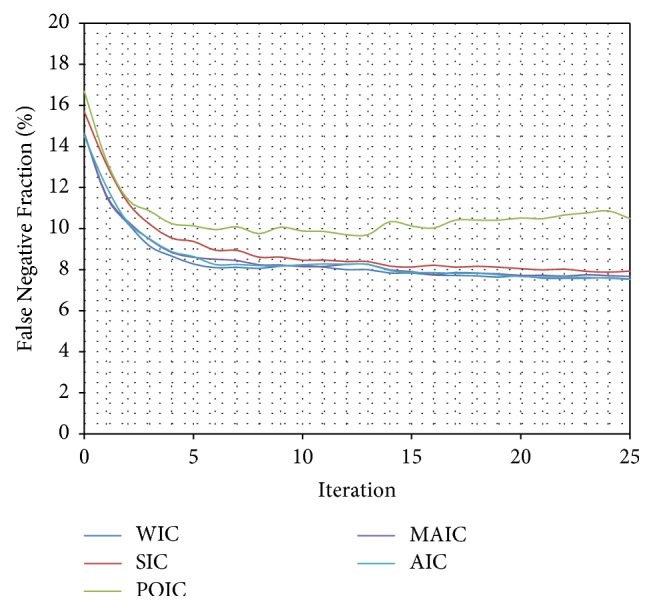
Comparison (percentage) of False Negative Fraction mean values for subsequent interactions of automatic segmentation for different optimization algorithms.

Only certain Inverse Compositional algorithms [38, 41–43, 48–52] were chosen to be tested: Wiberg Inverse Compositional (WIC) [38, 53–55] algorithm, Simultaneous Inverse Compositional (SIC) [49, 52] algorithm, Project-Out Inverse Compositional (POIC) [41] algorithm, Alternating Inverse Compositional (AIC) [42, 48] algorithm, and Modified Alternating Inverse Compositional (MAIC) [42, 48] algorithm. Three algorithms (WIC, AIC, and MAIC) achieved almost identical results (Table 1) (Figures 7, 8, and 9). Because of the best stability and lowest standard deviation (Table 1) Wiberg Inverse Compositional algorithm was chosen for further calculations.

What is more, to achieve reliable results, a mean value obtained from 100 procedure passes with 25 algorithm iterations each was computed and compared to results obtained manually by five experts (Table 2). The False Fraction is a general segmentation evaluation measure and is defined by the difference between manually segmented area and automatically segmented area, divided by the total area resulting from the manual segmentation. In this case the AAM algorithm (FF = 90.19 ± 1.01%) proved to be almost identical to expert 5, has almost the lowest standard deviation (*σ*_FF_ = 3.64%), and is almost unnoticeably worse than other experts. False Negative Fraction provides information about percentage of nonselected pixels classified by the investigators as a spine tissue and is an amount of manually segmented area not indicated by the automatic segmentation, divided by the total area resulting from the manual segmentation. The AAM has the highest False Negative Fraction (FNF = 7.72 ± 0.95%, *σ*_FNF_ = 3.42%) of all investigators. The True Positive Fraction provides information about percentage of properly segmented pixels and is an amount of automatically segmented area consistent with manual segmentation, divided by the total area resulting from the manual segmentation. The AAM method has the True Positive Fraction value of TPF = 92.28 ± 0.95% and standard deviation of *σ*_TPF_ = 3.42%.

**Figure 10:**
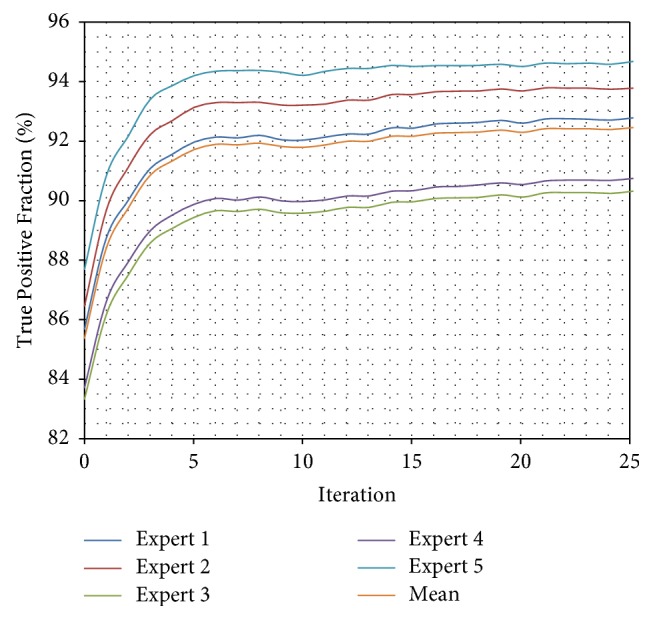
Comparison of True Positive Fraction for every iteration of automatic segmentation using presented method and manually segmented data from each expert. The True Positive Fraction provides information about percentage of properly segmented pixels. The TPF increases every iteration confirming proper functioning of presented algorithm—its convergence to manually segmented data.

**Figure 11:**
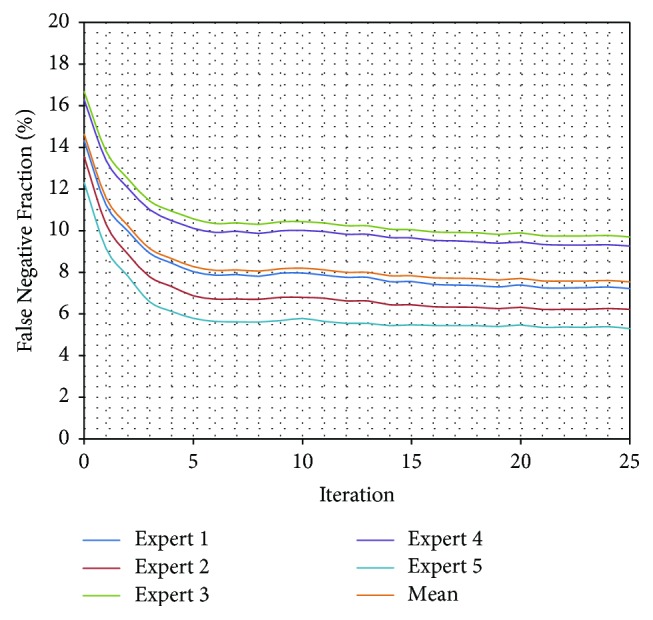
Comparison of False Negative Fraction for every iteration of automatic segmentation using presented method and manually segmented data from each expert. The FNF provides information about percentage of nonselected pixels classified by the investigators as a spine tissue. The FNF decreases every iteration confirming proper functioning of presented algorithm—its convergence to manually segmented data.

**Figure 12:**
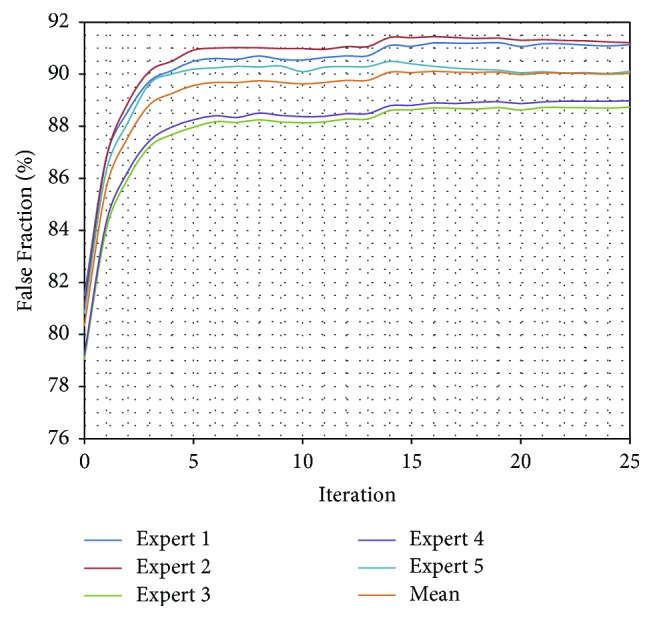
Comparison of False Fraction for every iteration of automatic segmentation using presented method and manually segmented data from each expert. The FF is a general segmentation evaluation measure. The FF increases every iteration confirming proper functioning of presented algorithm—its convergence to manually segmented data.

What is more, a test validating algorithm convergence by comparing automatic segmentation per iteration results with manual segmentation results was performed. A single algorithm pass with 25 iterations for a set of 50 previously unseen vertebrae images was executed and a change of TPF, FNF, and FF values for each iteration was calculated (Table 3). The True Positive Fraction increases every iteration (Figure 10), while the False Negative Fraction decreases (Figure 11) simultaneously leading the False Fraction to increase per iteration (Figure 12), confirming proper functioning of presented algorithm—its convergence to manually segmented data.

The Intraclass Correlation Coefficient (ICC) was calculated to evaluate the consistency of the vertebral bodies area determined by the experts and the computer (Tables 4 and 5). The high ICC results for single measurements ($\bar{r}=0.8336$) and for average measurements ($\bar{r}=0.9068$) with the *p* = 0.05 confirmed that the automatically and manually obtained segmentation results are comparable.

Additionally, the 10-fold cross-validation [56–58] analysis has been done. The database of 1000 training images was divided into equal parts and tested iteratively 10 times by training the model from 90% of images and performing a test on the remaining 10% with ground truth annotations. The ground truth landmarks have been used for shape interpolation using Catmull–Rom splines and compared with automatic segmentation results using TPF, FNF, and FF measures (Table 6). The False Fraction mean value of 91.37 ± 1.13% with standard deviation *σ*_FF_ = 5.76% confirmed a good model generalization to an independent dataset and resulting practical performance.

## 4. Discussion

Low resolution of presented data, high noise, and nonhomogeneous information about the tissues enforced the increasing of the quality of input data by initial filtration. From multiple interpolation methods [29, 59] widely used for image resampling, a high-resolution cubic spline has been chosen to increase the resolution of the input data, because of its good high-frequency response and high-frequency enhancement. What is more, a novel method of intensity inhomogeneity correction useful for sagittal MRI spine images has been presented. In last years, multiple methods used for intensity inhomogeneity correction emerged [60, 61]; however, they were mostly used for and tested with brain MRI scans. Because of a different application, segmentation of bone tissue instead of brain tissue, an additional method of initial filtration was developed.

For MRI images, a robust method for spine segmentation was prepared. The procedure combined well-known and widely tested Machine Learning methods [18]: Cascade of Boosted Classifiers [19–21] based on extended set Haar-like features [31] for preliminary vertebrae detection, with patch based Active Appearance Model [22, 23, 38] and Principal Component Analysis [39] for precise tissue segmentation. Usage of feature localization method and interpolation of the resulting information with centripetal Catmull–Rom splines [26, 27] omitted the problem of low quality. Due to the nature of Catmull–Rom splines [28] further optimization can be done to achieve better interpolation results. The paper [62] presents multiple recent methods for intervertebral disc segmentation, which can be treated as a similar task, including Machine Learning and deep learning based approaches. The segmentation results presented in the paper [62] were measured with dice overlap coefficients and varied from 81.6% to 92% for different methods. Comparing those results with obtained segmentation and generalization results of 90.19% and 91.37%, one can conclude that presented AAM approach provides a good segmentation performance and moreover can be applied for intervertebral discs localization and segmentation.

**Figure 13:**
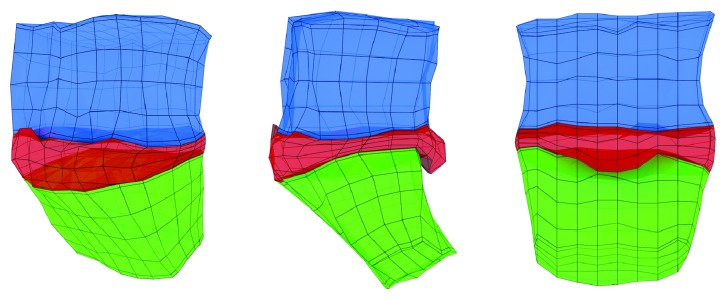
Discrete STL 3D model created manually from detected feature points (landmarks). The pathology of vertebrae and intervertebral disc is clearly visible.

**Figure 14:**
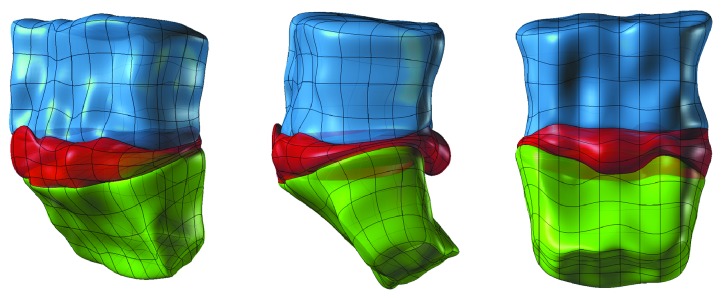
Continuous NURBS model created manually from detected feature points (landmarks), easily convertible to Finite Element mesh. The pathology of vertebrae and intervertebral disc is clearly visible.

In the future, automatically defined landmark localizations could be used for automatic creation of a discrete (Figure 13) and continuous (Figure 14) 3D spine models, which can be easily used in Finite Element Analysis [63–65], contrary to standard voxel representation.

Obtained three-dimensional model (Figures 13 and 14) contains information about the size and shape of the intervertebral disk and the adjacent vertebral bodies. Based on it, one can determine morphology of the intervertebral disk including direction, dimensions, and volume of the herniation of intervertebral disc, giving the clinicians a tool for better understanding of the pathology.

## Figures and Tables

**Table 1:** Comparison (percentage) of True Positive Fraction, False Negative Fraction, and False Fraction values and their standard deviations for automatically segmented data (significance level *α* = 0.05). *σ*_TPF_: standard deviation for True Positive Fraction, *σ*_FNF_: standard deviation for False Negative Fraction, *σ*_FF_: standard deviation for False Fraction.

Algorithm	TPF	FNF	FF	*σ*_TPF_	*σ*_FNF_	*σ*_FF_
WIC	92.46 ± 1.11	7.54 ± 1.11	90.04 ± 1.20	4.02	4.02	4.31
SIC	92.07 ± 1.23	7.93 ± 1.23	89.99 ± 1.30	4.45	4.45	4.69
POIC	89.51 ± 1.93	10.49 ± 1.93	85.89 ± 1.95	6.97	6.97	7.04
AIC	92.47 ± 1.10	7.53 ± 1.10	90.06 ± 1.21	3.95	3.95	4.37
MAIC	92.33 ± 1.10	7.67 ± 1.10	90.00 ± 1.20	3.97	3.97	4.33

**Table 2:** Comparison (percentage) of True Positive Fraction, False Negative Fraction, and False Fraction for automatically segmented data using presented method and manually segmented data from each expert (significance level *α* = 0.05). To achieve reliable results a mean value obtained from 100 procedure passes with 25 algorithm iterations each is presented. *σ*_TPF_: standard deviation for True Positive Fraction, *σ*_FNF_: standard deviation for False Negative Fraction, *σ*_FF_: standard deviation for False Fraction.

	TPF	FNF	FF	*σ*_TPF_	*σ*_FNF_	*σ*_FF_
Computer	92.28 ± 0.95	7.72 ± 0.95	90.19 ± 1.01	3.42	3.42	3.64
Expert 1	96.17 ± 0.92	3.83 ± 0.92	91.67 ± 1.27	3.32	3.32	4.58
Expert 2	95.13 ± 0.83	4.87 ± 0.83	92.11 ± 1	2.98	2.98	3.60
Expert 3	97.74 ± 0.43	2.26 ± 0.43	91.09 ± 1.33	1.56	1.56	4.78
Expert 4	97.56 ± 0.37	2.44 ± 0.37	91.49 ± 1.47	1.33	1.33	5.32
Expert 5	92.46 ± 1.33	7.54 ± 1.33	90.22 ± 1.31	4.81	4.81	4.74

**Table 3:** Comparison (percentage) of True Positive Fraction, False Negative Fraction, and False Fraction for automatically segmented data using presented method and manually segmented data from each expert.

Iteration	Expert 1			Expert 2			Expert 3			Expert 4			Expert 5			Mean		
	TPF	FNF	FF	TPF	FNF	FF	TPF	FNF	FF	TPF	FNF	FF	TPF	FNF	FF	TPF	FNF	FF
0	85.69	14.31	81.50	86.46	13.54	81.13	83.33	16.67	79.05	83.72	16.28	79.23	87.70	12.30	80.81	85.38	14.62	80.34
1	88.76	11.24	86.81	89.69	10.31	86.76	86.18	13.82	84.03	86.62	13.38	84.31	90.86	9.14	86.28	88.42	11.58	85.64
2	90.02	9.98	88.58	91.13	8.87	88.91	87.51	12.49	86.00	87.95	12.05	86.27	92.18	7.82	88.15	89.76	10.24	87.58
3	91.08	8.92	89.74	92.22	7.78	90.13	88.58	11.42	87.23	89.00	11.00	87.45	93.42	6.58	89.65	90.86	9.14	88.84
4	91.56	8.44	90.14	92.68	7.32	90.50	89.06	10.94	87.68	89.52	10.48	87.97	93.87	6.13	90.00	91.34	8.66	89.26
5	91.97	8.03	90.51	93.13	6.87	90.93	89.43	10.57	87.97	89.89	10.11	88.25	94.21	5.79	90.20	91.73	8.27	89.57
6	92.14	7.86	90.61	93.29	6.71	91.00	89.66	10.34	88.18	90.08	9.92	88.41	94.36	5.64	90.24	91.91	8.09	89.69
7	92.11	7.89	90.58	93.28	6.72	91.02	89.63	10.37	88.15	90.03	9.97	88.34	94.38	5.62	90.31	91.89	8.11	89.68
8	92.19	7.81	90.70	93.30	6.70	91.02	89.70	10.30	88.25	90.13	9.87	88.51	94.39	5.61	90.28	91.94	8.06	89.75
9	92.04	7.96	90.57	93.20	6.80	90.99	89.58	10.42	88.16	90.01	9.99	88.42	94.32	5.68	90.32	91.83	8.17	89.69
10	92.03	7.97	90.54	93.20	6.80	90.99	89.56	10.44	88.14	89.99	10.01	88.37	94.22	5.78	90.10	91.80	8.20	89.63
11	92.14	7.86	90.64	93.24	6.76	90.95	89.63	10.37	88.17	90.05	9.95	88.38	94.36	5.64	90.27	91.88	8.12	89.68
12	92.24	7.76	90.71	93.37	6.63	91.06	89.77	10.23	88.28	90.17	9.83	88.49	94.45	5.55	90.30	92.00	8.00	89.77
13	92.24	7.76	90.71	93.37	6.63	91.06	89.77	10.23	88.28	90.17	9.83	88.49	94.45	5.55	90.30	92.00	8.00	89.77
14	92.45	7.55	91.11	93.56	6.44	91.42	89.93	10.07	88.61	90.33	9.67	88.79	94.56	5.44	90.49	92.17	7.83	90.08
15	92.44	7.56	91.07	93.56	6.44	91.40	89.95	10.05	88.63	90.35	9.65	88.80	94.52	5.48	90.40	92.17	7.83	90.06
16	92.58	7.42	91.21	93.65	6.35	91.45	90.06	9.94	88.71	90.47	9.53	88.90	94.55	5.45	90.31	92.26	7.74	90.11
17	92.61	7.39	91.19	93.67	6.33	91.41	90.09	9.91	88.69	90.49	9.51	88.87	94.56	5.44	90.23	92.28	7.72	90.08
18	92.64	7.36	91.19	93.68	6.32	91.37	90.10	9.90	88.66	90.54	9.46	88.92	94.56	5.44	90.19	92.31	7.69	90.07
19	92.70	7.30	91.21	93.75	6.25	91.39	90.18	9.82	88.72	90.60	9.40	88.94	94.60	5.40	90.16	92.37	7.63	90.09
20	92.61	7.39	91.07	93.68	6.32	91.30	90.11	9.89	88.62	90.55	9.45	88.87	94.53	5.47	90.05	92.30	7.70	89.98
21	92.75	7.25	91.17	93.78	6.22	91.33	90.25	9.75	88.73	90.67	9.33	88.94	94.65	5.35	90.10	92.42	7.58	90.05
22	92.75	7.25	91.17	93.78	6.22	91.30	90.26	9.74	88.72	90.69	9.31	88.97	94.63	5.37	90.05	92.42	7.58	90.04
23	92.74	7.26	91.12	93.78	6.22	91.28	90.25	9.75	88.71	90.69	9.31	88.96	94.64	5.36	90.07	92.42	7.58	90.03
24	92.71	7.29	91.09	93.74	6.26	91.24	90.23	9.77	88.70	90.68	9.32	88.96	94.61	5.39	90.03	92.39	7.61	90.00
25	92.78	7.22	91.14	93.78	6.22	91.21	90.30	9.70	88.74	90.74	9.26	88.98	94.70	5.30	90.11	92.46	7.54	90.04

**Table 4:** The Intraclass Correlation Coefficient (ICC) of the vertebral bodies area determined by experts and computer, for single measurements with the *p* = 0.05.

	Computer	Expert 1	Expert 2	Expert 3	Expert 4	Expert 5
Computer		0.8815	0.9153	0.7368	0.7210	0.9134
Expert 1	0.8815		0.9442	0.8761	0.8622	0.8685
Expert 2	0.9153	0.9442		0.8721	0.8695	0.9266
Expert 3	0.7368	0.8761	0.8721		0.9586	0.7830
Expert 4	0.7210	0.8622	0.8695	0.9586		0.7670
Expert 5	0.9134	0.8685	0.9266	0.7830	0.7670	

**Table 5:** The Intraclass Correlation Coefficient (ICC) of the vertebral bodies area determined by experts and computer, for average measurements with the *p* = 0.05.

	Computer	Expert 1	Expert 2	Expert 3	Expert 4	Expert 5
Computer		0.9370	0.9558	0.8485	0.8379	0.9547
Expert 1	0.9370		0.9713	0.9339	0.9260	0.9296
Expert 2	0.9558	0.9713		0.9317	0.9302	0.9619
Expert 3	0.8485	0.9339	0.9317		0.9789	0.8783
Expert 4	0.8379	0.9260	0.9302	0.9789		0.8681
Expert 5	0.9547	0.9296	0.9619	0.8783	0.8681	

**Table 6:** 10-fold cross-validation comparison (percentage) of True Positive Fraction (TPF), False Negative Fraction (FNF), and False Fraction (FF) for automatically segmented data using presented method and ground truth annotations (significance level *α* = 0.05). *σ*_TPF_: standard deviation for True Positive Fraction, *σ*_FNF_: standard deviation for False Negative Fraction, *σ*_FF_: standard deviation for False Fraction.

*k*-fold	TPF	FNF	FF	*σ*_TPF_	*σ*_FNF_	*σ*_FF_
1	95.72 ± 1.27	4.28 ± 1.27	90.12 ± 1.38	6.50	6.50	7.02
2	97.39 ± 0.77	2.61 ± 0.77	91.29 ± 1.07	3.91	3.91	5.47
3	95.73 ± 0.86	4.27 ± 0.86	91.18 ± 1.72	4.41	4.41	8.75
4	95.13 ± 0.85	4.87 ± 0.85	91.98 ± 0.98	4.35	4.35	5.00
5	95.00 ± 0.85	5.00 ± 0.85	91.32 ± 1.01	4.32	4.32	5.14
6	96.18 ± 0.76	3.82 ± 0.76	90.87 ± 0.94	3.88	3.88	4.80
7	96.35 ± 0.82	3.65 ± 0.82	92.13 ± 1.06	4.17	4.17	5.42
8	96.14 ± 0.69	3.86 ± 0.69	92.20 ± 0.95	3.50	3.50	4.82
9	96.03 ± 0.71	3.97 ± 0.71	92.09 ± 0.98	3.64	3.64	5.02
10	93.56 ± 1.10	6.44 ± 1.10	90.53 ± 1.21	5.60	5.60	6.17

Mean	95.72 ± 0.87	4.28 ± 0.87	91.37 ± 1.13	4.43	4.43	5.76

## References

[1] Finch P. (2006). Technology insight: imaging of low back pain.

[2] Airaksinen O., Brox J. I., Cedraschi C. (2006). Chapter 4: european guidelines for the management of chronic nonspecific low back pain.

[3] Schoenfeld A. J., Weiner B. K. (2010). Treatment of lumbar disc herniation: evidence-based practice.

[4] Yang H., Liu H., Zemin L. (2015). Low back pain associated with lumbar disc herniation: role of moderately degenerative disc and annulus fibrous tears.

[5] Lawrence R. C., Helmick C. G., Arnett F. C. (1998). Estimates of the prevalence of arthritis and selected musculoskeletal disorders in the United States.

[6] Eun S. S., Lee H.-Y., Lee S.-H., Kim K. H., Liu W. C. (2012). MRI versus CT for the diagnosis of lumbar spinal stenosis.

[7] Ros L., Mota J., Guedea A., Bidgood D. (1998). Quantitative measurements of the spinal cord and canal by MR imaging and myelography.

[8] Dong X., Zheng G. (2016). Automated 3D lumbar intervertebral disc segmentation from MRI data sets.

[9] Schmidt S., Kappes J., Bergtholdt M. (2007). Spine detection and labeling using a parts-based graphical model.

[10] Corso J. J., Alomari R. S., Chaudhary V. (2008). Lumbar disc localization and labeling with a probabilistic model on both pixel and object features.

[11] Chevrefils C., Cheriet F., Aubin C.-É., Grimard G. (2009). Texture analysis for automatic segmentation of intervertebral disks of scoliotic spines from MR images.

[12] Michopoulou S. K., Costaridou L., Panagiotopoulos E., Speller R., Panayiotakis G., Todd-Pokropek A. (2009). Atlas-based segmentation of degenerated lumbar intervertebral discs from MR images of the spine.

[13] Ismail B. A., Punithakumar K., Gregory G., Romano W., Shuo L. Graph cuts with invariant object-interaction priors: application to intervertebral disc segmentation.

[14] Neubert A., Fripp J., Engstrom C. (2012). Automated detection, 3D segmentation and analysis of high resolution spine MR images using statistical shape models.

[15] Law M. W. K., Tay K., Leung A., Garvin G. J., Li S. (2013). Intervertebral disc segmentation in MR images using anisotropic oriented flux.

[16] Glocker B., Zikic D., Konukoglu E., Haynor D. R., Criminisi A. Vertebrae localization in pathological spine CT via dense classification from sparse annotations.

[17] Cortes C., Vapnik V. (1995). Support-vector networks.

[18] Theodoridis S. (2015).

[19] Hastie T., Tibshirani R., Friedman J. (2001).

[20] Friedman J., Hastie T., Tibshirani R. (2000). Additive logistic regression: a statistical view of boosting.

[21] Viola P., Jones M. Rapid object detection using a boosted cascade of simple features.

[22] Alabort-I-Medina J., Antonakos E., Booth J., Snape P., Zafeiriou S. Menpo: a comprehensive platform for parametric image alignment and visual deformable models.

[23] Edwards G. J., Taylor C. J., Cootes T. F. Interpreting face images using active appearance models.

[24] Jolliffe I. (2002).

[25] Bradski G. (2000). The opencv library.

[26] Barry P. J., Goldman R. N. (1988). A recursive evaluation algorithm for a class of Catmull-Rom splines.

[27] Catmull E., Raphael R. (1974). A class of local interpolating splines.

[28] Yuksel C., Schaefer S., Keyser J. (2011). Parameterization and applications of CatmullRom curves.

[29] Parker J. A., Kenyon R. V., Troxel D. E. (1983). Comparison of Interpolating Methods for Image Resampling.

[30] Catté F., Lions P.-L., Morel J.-M., Coll T. (1992). Image selective smoothing and edge detection by nonlinear diffusion.

[31] Lienhart R., Maydt J. An extended set of Haar-like features for rapid object detection.

[32] Bookstein F. L. (1989). Principal warps: thin-plate splines and the decomposition of deformations.

[33] Rohr K., Stiehl H. S., Sprengel R., Buzug T. M., Weese J., Kuhn M. H. (2001). Landmark-based elastic registration using approximating thin-plate splines.

[34] Telea A. (2004). An image inpainting technique based on the fast marching method.

[35] Freund Y., Schapire R. E. A desicion-theoretic generalization of on-line learning and an application to boosting.

[36] Amit Y., Geman D., Wilder K. (1997). Joint induction of shape features and tree classifiers.

[37] Viola P., Jones M. J. (2004). Robust real-time face detection.

[38] Alabort-i-Medina J., Zafeiriou S. (2017). A unified framework for compositional fitting of active appearance models.

[39] Abdi H., Williams L. J. (2010). Principal component analysis.

[40] Lucas B. D., Kanade T. An iterative image registration technique with an application to stereo vision.

[41] Matthews J., Baker S. (2004). Active appearance models revisited.

[42] Papandreou G., Maragos P. Adaptive and constrained algorithms for inverse compositional active appearance model fitting.

[43] Tzimiropoulos G., Pantic M. Optimization problems for fast AAM fitting in-the-wild.

[44] Tzimiropoulos G., Pantic M. Gauss-Newton deformable part models for face alignment in-the-wild.

[45] Fawcett T. (2006). An introduction to ROC analysis.

[46] Fenster A., Chiu B. Evaluation of Segmentation algorithms for Medical Imaging.

[47] Metz C. E. (1986). Special articles roc methodology in radiologic imaging.

[48] Alabort-I-Medina J., Zafeiriou S. Bayesian active appearance models.

[49] Baker S., Gross R., Matthews I. Lucas-kanade 20 years on: a unifying framework.

[50] Baker S., Matthews I. Equivalence and efficiency of image alignment algorithms.

[51] Baker S., Matthews I. (2004). Lucas-Kanade 20 years on: a unifying framework.

[52] Gross R., Matthews I., Baker S. (2005). Generic vs. person specific active appearance models.

[53] Okatani T., Deguchi K. (2007). On the wiberg algorithm for matrix factorization in the presence of missing components.

[54] Strelow D. General and nested wiberg minimization.

[55] Wiberg T. Computation of principal components when data are missing.

[56] Devijver P. A., Kittler J. (1982).

[57] Geisser S. (1993).

[58] Kohavi R. A study of cross-validation and bootstrap for accuracy estimation and model selection.

[59] Lehmann T. M., Gönner C., Spitzer K. (1999). Survey: interpolation methods in medical image processing.

[60] Hou Z. (2006). A review on MR image intensity inhomogeneity correction.

[61] Vovk U., Pernuš F., Likar B. (2007). A review of methods for correction of intensity inhomogeneity in MRI.

[62] Zheng G., Chu C., Belavý D. L. (2017). Evaluation and comparison of 3D intervertebral disc localization and segmentation methods for 3D T2 MR data: A grand challenge.

[63] Hughes T. J. R. (2012).

[64] Szabo B. A., Babuška I. (1991).

[65] Zienkiewicz O. C., Taylor R. L., Taylor R. L. (1977).

